# Association between risk factors and the occurrence of bovine tuberculosis in cattle and buffalo herds: evidence from a multivariate analysis (2017–2023)—Campania regione

**DOI:** 10.3389/fvets.2026.1781339

**Published:** 2026-03-09

**Authors:** Federica Gargano, Roberta Brunetti, Marco Tamba, Giorgio Galletti, Loredana Baldi, Esterina De Carlo, Orlando Paciello, Giuseppe Iovane, Maria Ottaiano

**Affiliations:** 1 Istituto Zooprofilattico Sperimentale del Mezzogiorno, Portici, Italy; 2 Istituto Zooprofilattico Sperimentale della Lombardia e dell Emilia-Romagna Bruno Ubertini, Brescia, Italy

**Keywords:** bovine, bufalin, logit analysis, risk factor, tuberculosis

## Abstract

Bovine tuberculosis (bTB), caused by *Mycobacterium bovis*, is one of the most important zoonotic diseases of veterinary and public health concern, with significant economic, productive, and sanitary implications. In the Campania region, characterized by a high density of cattle and buffalo farms and a complex diversity of production systems, understanding the risk factors that influence the persistence and spread of the disease is essential for designing effective control and eradication strategies. This retrospective longitudinal observational cohort study analyzed a seven-year period (2017–2023) with the aim of identifying the main structural, management, and environmental variables associated with the occurrence of bTB outbreaks in cattle and buffalo herds across Campania. Data were integrated from national information systems (SANAN, SIMAN, and BDN) and analyzed using Fisher's exact test, Wilcoxon test, and univariate and multivariate logistic regression models. The covariates considered included herd size, animal movements, previous history of infection, territorial characteristics (farm density, outbreak proximity, presence of pasture), and the presence of other domestic species. In the buffalo cohort (*n* = 892), 13% of farms reported at least one positive case. Independent risk factors identified were previous infection (OR = 3.14; 95% CI: 1.61–5.92), introduction of animals from outside the region (OR = 0.59; 95% CI: 0.19–0.85), and the number of outbreaks within a 2 km radius (OR = 1.19; 95% CI: 1.07–1.33). In the cattle cohort (*n* = 4,467), the prevalence of positivity was 3%. Significant predictors included previous infection (OR = 8.98; 95% CI: 4.18–17.76), presence of pasture within 2 km (OR = 2.65; 95% CI: 1.62–4.19), animal movements (OR = 1.91; 95% CI: 1.11–3.57), and the number of outbreaks within a 2 km buffer (OR = 1.43; 95% CI: 1.28–1.58). The findings highlight the crucial role of herd-level (size and movements), historical (previous infection), and spatial (outbreak proximity, pasture presence) factors in the transmission dynamics of bTB. The integration of epidemiological and spatial analytical approaches provides a comprehensive understanding of the regional risk landscape, offering valuable insights for improving biosecurity, surveillance, and control strategies aimed at reducing the burden of bovine tuberculosis in both cattle and buffalo populations.

## Introduction

Bovine tuberculosis (bTB) is one of the most important zoonotic diseases of veterinary and public health concern, due to its complex epidemiology and significant economic, productive, and health implications. The disease, caused by *Mycobacterium bovis*, continues to represent a major challenge for livestock production systems worldwide, affecting animal welfare, trade, and food safety (1–8). In regions such as Campania, where cattle and buffalo farming is widespread and characterized by considerable production heterogeneity, identifying the factors that influence the persistence and spread of bTB within and between farms is crucial to guide increasingly effective control and eradication strategies.

This retrospective longitudinal observational study, conducted over a seven-year period (2017–2023), aimed to identify the main structural, management, and environmental variables associated with the occurrence of bTB outbreaks in livestock holdings across the Campania region. The analysis considered two distinct cohorts: one consisting of buffalo farms and the other of cattle farms, both selected according to strict inclusion criteria to ensure data consistency and the reliability of statistical results.

By integrating data from several national information systems—including SANAN (National Animal Health Information System), SIMAN (Animal Disease Information System), and the National Database for Animal Identification and Registration (BDN)—a comprehensive and detailed database was constructed, encompassing both farm-level and territorial characteristics. Advanced statistical and spatial modeling techniques were applied to estimate the effects of multiple covariates, such as herd size (3, 4, 6), intensity and direction of animal movements (1, 4), history of previous infection, environmental features of the surrounding area (e.g., land use, farm density, wildlife interface) (4, 7), and the presence of other domestic animal species (2, 5, 8).

The combined evaluation of these factors allowed for a deeper understanding of the multifactorial dynamics that drive bTB transmission within the regional livestock context. The results provide concrete, evidence-based insights to support the improvement of surveillance, prevention, and control strategies at the territorial level, contributing to the progressive reduction of the disease's incidence in both buffalo and cattle populations.

## Study design

A retrospective longitudinal cohort observational study was conducted to evaluate the association between specific risk factors and the occurrence of bovine tuberculosis (bTB) in cattle and buffalo farms located in the Campania Region. The cohort included farms with similar structural and management characteristics, differing only in their exposure to the investigated risk factors.

### Farm selection criteria

#### Inclusion criteria

- Farms located in the Campania Region;
- Presence of cattle or buffalo;
- Annual official bTB testing performed for each year of the 2017–2023 period;
- Breeding-oriented production system.

#### Exclusion criteria

- Farms located outside the Campania Region;
- Farms without cattle and/or buffalo;
- Farms not subjected to official bTB testing for all seven years considered;
- Farms with a fattening-oriented production system.

### Observation period and data sources

The observation period covered seven years (2017–2023). Data were obtained from three main national information systems:

- SANAN (Animal Health Information System): used to collect the results of official health inspections on farms;
- SIMAN (Notifiable Animal Disease Information System): used to identify and confirm bTB outbreaks;
- BDN (National Livestock Database): used to obtain information on farm location, herd size, and animal movements.

### Data cleaning and management

Data extraction, cleaning, and management were carried out using Microsoft Access (Office suite) and R-Studio version 4.4.1. The identification of the study cohort followed these steps:

- Extraction from SANAN of farms subjected to annual testing (2017–2023);
- Exclusion of farms not present in all years of the observation period;
- Extraction of confirmed outbreaks from SIMAN (2017–2023);
- Cross-referencing SANAN and SIMAN data for integration and verification.

### Covariate construction

The following explanatory covariates were constructed, separately for the two cohorts (cattle and buffalo):

- Production type: categorical variable: Dairy, Beef, Mixed;
- Previous positivity (within 5 years prior to outbreak confirmation): dichotomous variable Yes/No;
- Average herd size during the period: annual average number of animals as of December 31 of each year;
- Presence of small ruminants (sheep/goats): dichotomous variable Yes/No;
- Animal movements:
  ° Dichotomous variable indicating presence/absence of at least one movement during the period;
  ° Median number of animals moved during the observation period;

### Origin of introduced animals

° From bTB-free/non-free zones;
° From within-region/outside-region (in cases of dual origin, the farm was classified as “outside-region”);

- Number of farms (active at least once during the study period) within a 2 km buffer of the cohort farms;
- Number of outbreaks within a 2 km buffer of the cohort farms;
- Presence of pasture within the 2 km buffer.

### Statistical analysis

The collected variables were initially processed according to their nature. For qualitative variables, absolute and relative frequencies were calculated, while for quantitative variables, basic descriptive statistics such as mean, median, and standard deviation were computed to summarize the data distribution.

To assess the association between each covariate and the outcome variable (presence or absence of bovine tuberculosis), Fisher's exact test was used for qualitative variables, and the non-parametric Wilcoxon test was applied for quantitative variables. The statistical significance threshold was set at *p* < 0.05.

Subsequently, logistic regression models were constructed for each cohort (cattle and buffalo). In the presence of correlated covariates, univariate analyses were performed. In the absence of such correlations, multivariate logistic regression models were developed to estimate the independent effect of each risk factor on the outcome.

## Results

### Buffalo cohort

The buffalo cohort consisted of a total of 892 farms subjected to tuberculosis testing in the Campania Region during the 2017–2023 period. Among these, 13% had at least one positive result, while 87% tested negative throughout the entire period.

The association between the outcome (positive/negative) and each covariate (Table 1) was assessed using the Wilcoxon test for quantitative variables and Fisher's exact test for qualitative variables.

**Table 1:** Fisher's exact test and Wilcoxon test—Buffalo species.

**Variables**	***N* (*N* = 775)**	***P* (*N* = 117)**	***P*-value**
**The average herd size during the period**			
Mean (SD)	294 (248)	385 (415)	0.0163
Median [Min, Max]	226 [1.00, 1,660]	269 [25.0, 2,780]	
**Median number of animals moved (during the period)**			
Mean (SD)	58.6 (110)	85.7 (141)	0.0202
Median [Min, Max]	16.0 [1.00, 803]	32.5 [1.00, 806]	
Missing	200 (25.8%)	28 (23.9%)	
**Number of farms within a 2 km buffer**			
Mean (SD)	15.0 (11.3)	17.6 (11.8)	0.0183
Median [Min, Max]	13.0 [0, 43.0]	15.0 [0, 43.0]	
**Number of outbreaks within a 2 km buffer**			
Mean (SD)	1.44 (1.96)	2.23 (1.88)	<0.001
Median [Min, Max]	1.00 [0, 9.00]	2.00 [0, 7.00]	
**Previous positivity**			
No	731 (94.3%)	90 (76.9%)	<0.001
Yes	44 (5.7%)	27 (23.1%)	
**Presence of pasture within a 2 km buffer**			
No	606 (78.2%)	107 (91.5%)	<0.001
Yes	169 (21.8%)	10 (8.5%)	
**Origin of animal movements**			
Out of region	187 (24.1%)	44 (37.6%)	0.00264
Within region	388 (50.1%)	45 (38.5%)	
Missing	200 (25.8%)	28 (23.9%)	
**Origin status**			
Non-disease-free	573 (73.9%)	84 (71.8%)	<0.001
Disease-free	2 (0.3%)	5 (4.3%)	
Missing	200 (25.8%)	28 (23.9%)	
**Presence of sheep and goats**			
No	715 (92.3%)	114 (97.4%)	0.0502
Yes	60 (7.7%)	3 (2.6%)	
**Movements**			
No	200 (25.8%)	28 (23.9%)	0.734
Yes	575 (74.2%)	89 (76.1%)	

The following variables were found to be significantly associated with the outcome (p-value < 0.05):

Average number of heads during the period;

Median number of animals moved;

Previous positivity;

Presence of small ruminants (sheep/goats);

Origin of animal movements;

Health status of the area of origin;

Number of farms within the 2 km buffer;

Number of outbreaks within the 2 km buffer;

Presence of pasture within the 2 km buffer.

Preliminary correlation analysis revealed a statistically significant relationship between the number of heads present at the end of the period and the median number of animals moved, as detected by Spearman's test (*p* < 0.05). To avoid multicollinearity issues, separate univariate logistic regression models were constructed for each of these variables. In the first univariate model, the average number of heads at the end of the period, after logarithmic transformation, was associated with an increased probability of tuberculosis positivity: the odds ratio (OR) was 1.37 (95% CI: 1.09–1.74), indicating a 37% increase in risk for each unit increase in the log-transformed variable. Similarly, the median number of animals moved, also log-transformed, was significantly associated with the outcome, with an OR of 1.16 (95% CI: 1.01–1.33), suggesting a 16% increase in the probability of positivity. In the subsequent multivariate model (Table 2), several independent risk factors emerged. Previous positivity in the preceding years confirmed itself as one of the main predictors, with an OR of 3.14 (95% CI: 1.61–5.92), indicating more than a threefold increased risk in farms with a history of outbreaks. Conversely, the presence of small ruminants (sheep/goats) was associated with a protective effect, with an OR of 0.25 (95% CI: 0.04–0.91), although this was not statistically significant. An unexpected finding concerns the health status of the area of origin of the introduced animals: movements originating from non–disease-free zones showed an association with a lower risk of positivity (OR = 0.07; 95% CI: 0.01–0.40), although this counterintuitive result warrants further investigation. The geographic origin of introduced animals was also relevant: farms introducing animals exclusively from within the same region had a significantly lower risk compared to those also introducing animals from outside the region (OR = 0.59; 95% CI: 0.19–0.85).

**Table 2:** Logistic regression model.

**Variable**	**Odds ratio**	**std.error**	**statistic**	***P*-value**	**IC 95% (Inf)**	**IC 95% (Sup)**
Intercept	2.800	0.8877970	1.159648	0.246	0.553	21.566
Previous positivity = “Yes”	3.138	0.3300287	3.465213	**0.001**	1.611	5.918
Presence of small ruminants (ovi-caprini) = “Yes”	0.257	0.7715117	−1.763118	0.078	0.039	0.914
Health status of origin = “Non disease-free”	0.077	0.9038761	−2.838960	**0.005**	0.010	0.403
Origin of movements = “Within region”	0.591	0.2415652	−2.179831	**0.029**	0.368	0.951
Presence of pasture in 2 km buffer = “Yes”	0.434	0.3711403	−2.250832	**0.024**	0.196	0.855

Bold values indicate statistically significant results (*p* < 0.05).

Finally, an additional multivariate model was constructed (Table 3) including spatial covariates related to the 2 km buffer around the farms. In this model, the number of outbreaks present within the buffer was significantly associated with the outcome, with an OR of 1.19 (95% CI: 1.07–1.33), indicating an 18.8% increase in the risk of positivity for each additional nearby outbreak. Conversely, the number of farms within the buffer did not show a significant effect (OR = 1.002), as also confirmed by the confidence interval including unity. No multicollinearity was observed between these two variables (VIF < 5), but only the number of outbreaks contributed significantly to the model, as confirmed by the deviance analysis (ANOVA). It should also be noted that, although the presence of pasture within the buffer was statistically significant and showed a protective effect in the preliminary analysis, this variable was not included in the final models for the buffalo cohort.

**Table 3:** Multivariate logistic regression model.

**Variable**	**Odds ratio**	**std.error**	**statistic**	***P*-value**	**IC 95% (Inf)**	**IC 95% (Sup)**
Intercept	0.108	0.17702466	−12.5910307	**0.000**	0.075	0.151
Number of farms within a 2 km buffer = “Yes”	1.002	0.01060303	0.1526352	0.879	0.981	1.022
Number of outbreaks within a 2 km buffer = “Yes”	1.188	0.05575368	3.0942332	**0.002**	1.065	1.326

Bold values indicate statistically significant results (*p* < 0.05).

### Cattle cohort

The cattle cohort consists of 4,467 farms monitored for tuberculosis between 2017 and 2023 in the Campania Region. Of these, 3% had at least one positive outcome, while the remaining 97% tested negative. Similarly, the association between the outcome variable and the covariates was assessed using the Wilcoxon test for quantitative variables and Fisher's exact test for qualitative variables (Table 4).

**Table 4:** Fisher's and Wilcoxon's tests—bovine species.

**Variables**	***N* (*N* = 4,349)**	***P* (*N =* 118)**	***P*-value**
**Average number of heads during the period**			
Mean (SD)	40.9 (110)	83.0 (207)	<0.001
Median [Min, Max]	18.0 [1.00, 3,870]	35.0 [3.00, 1,460]	
**Median number of animals moved**			
Mean (SD)	11.1 (106)	20.6 (45.9)	<0.001
Median [Min, Max]	3.00 [1.00, 5,280]	4.25 [1.00, 268]	
Missing	889 (20.4%)	14 (11.9%)	
**Number of farms within a 2 km buffer**			
Mean (SD)	31.0 (32.1)	24.8 (20.9)	0.023
Median [Min, Max]	21.0 [0, 322]	17.0 [2.00, 97.0]	
**Number of outbreaks within a 2 km buffer**			
Mean (SD)	0.285 (0.834)	0.966 (1.72)	<0.001
Median [Min, Max]	0 [0, 9.00]	0 [0, 7.00]	
**Previous positivity**			
No	4,302 (98.9%)	107 (90.7%)	<0.001
Yes	47 (1.1%)	11 (9.3%)	
**Production orientation**			
Meat	1,974 (45.4%)	60 (50.8%)	0.268
Milk	619 (14.2%)	19 (16.1%)	
Mixed	1,751 (40.3%)	39 (33.1%)	
Missing	5 (0.1%)	0 (0%)	
**Origin of animal movements**			
Both	1,302 (29.9%)	47 (39.8%)	0.272
Out of region	100 (2.3%)	3 (2.5%)	
In region	2,058 (47.3%)	54 (45.8%)	
Missing	889 (20.4%)	14 (11.9%)	
**Status provenienza**			
Disease-free	15 (0.3%)	0 (0%)	1
Non-disease-free	3,441 (79.1%)	104 (88.1%)	
Missing	893 (20.5%)	14 (11.9%)	
**Presence of sheep and goats**			
No	889 (20.4%)	14 (11.9%)	0.02
Yes	3,460 (79.6%)	104 (88.1%)	
**Presence of pasture within a 2 km buffer**			
No	3,962 (91.1%)	94 (79.7%)	<0.001
Yes	387 (8.9%)	24 (20.3%)	

The variables found to be associated with the study outcome with a p-value < 0.05 are:

Average number of heads during the period;

Median number of heads moved;

Previous positivity;

Presence of sheep and goats;

Number of farms within a 2 km buffer;

Number of outbreaks within a 2 km buffer;

Presence of pasture within a 2 km buffer.

To evaluate the association between certain quantitative variables and the study outcome, univariate logistic regression models were constructed. This approach was chosen because the considered variables were correlated with each other, and their simultaneous inclusion in a single multivariate model could have caused multicollinearity issues, compromising the estimation and interpretation of effects.

Specifically, the median number of movements (log-transformed to improve linearity and normality of the distribution) was found to be significantly associated with the outcome, with an odds ratio (OR) of 1.50 (95% confidence interval [CI]: 1.17–1.89). This result indicates that an increase in the median number of movements is associated with a 50% increase in the risk of the event under study. Similarly, the average number of heads present at the end of the period (also log-transformed) showed a statistically significant association, with an OR of 1.56 (95% CI: 1.36–1.80). This suggests that as the average number of heads increases, the risk rises by 56%, with a stable and well-defined effect.

The multivariate logistic regression analysis (Table 5) highlighted several variables significantly associated with the probability of disease. In particular, previous positivity represents a strong predictor of the outcome: animals coming from farms with prior positivity have nearly nine times higher odds of disease compared to those without previous positivity (OR = 8.98; 95% CI: 4.18–17.76), confirming the importance of historical factors in infection dynamics.

**Table 5:** Multivariate logistic regression model—bovine species.

**Variable**	**Odds ratio**	**Std.error**	**Statistic**	***P*-value**	**IC 95% (Inf)**	**IC 95% (Sup)**
Intercept	0.010	0.28698757	-15.889644	**0.000**	0.006	0.018
Previous positivity = “Yes”	8.980	0.36623113	5.993402	**0.000**	4.175	17.761
Presence of pasture within 2 km buffer = “Yes”	2.653	0.24169332	4.036748	**0.000**	1.619	4.192
Movements = “Yes”	1.910	0.29631375	2.184588	**0.029**	1.107	3.567
Number of outbreaks within 2 km buffer = “Yes”	1.425	0.05268297	6.724652	**0.000**	1.279	1.575

Bold values indicate statistically significant results (*p* < 0.05).

The presence of pasture within 2 km of the farm was also significantly associated with the outcome, with an approximately 2.65-fold increase in odds (OR = 2.65; 95% CI: 1.62–4.19), suggesting a possible role of environmental or inter-farm interactions in transmission.

The variable related to movements shows a significant but moderate association: farms with movements have nearly double the odds of disease compared to those without (OR = 1.91; 95% CI: 1.11–3.57), emphasizing the importance of health control in animal flows.

Finally, the presence of outbreaks within a 2 km radius represents an additional risk factor: for each outbreak present within the buffer, there is a 43% increase in the odds (OR = 1.43; 95% CI: 1.28–1.58), supporting the hypothesis of local spatial spread of the disease. All estimated effects are statistically significant (*p* < 0.05), with narrow confidence intervals indicating precise and robust effect estimates.

## Conclusion

The findings of this study provide strong evidence of multiple risk factors significantly associated with the occurrence of bovine tuberculosis (bTB) in both buffalo and cattle farms in the Campania region. The consistent associations observed across the two species highlight the multifactorial nature of bTB epidemiology (2, 3, 5, 8) and emphasize the interplay between herd-level, management, and environmental determinants in sustaining infection within endemic areas.

Among the most influential variables, herd size (3, 4, 6) and the volume of animal movements (1, 4) emerged as major contributors to the probability of infection. Larger herds, characterized by higher animal density and more frequent trading activities, likely facilitate both intra- and inter-farm transmission through increased contact opportunities and complex movement networks. These results underscore the importance of implementing stringent movement controls and enhancing traceability systems as key components of disease control strategies.

Previous infection history was confirmed as one of the strongest predictors of risk in both species (6, 7), suggesting that persistence of infection within herds remains a critical obstacle to eradication. This finding points to potential gaps in test-and-slaughter policies, biosecurity enforcement, or environmental persistence of *Mycobacterium bovis* (5, 8), all of which may contribute to disease re-emergence over time. Furthermore, the presence of outbreaks within a 2 km radius of a farm was consistently identified as a significant risk indicator, supporting the hypothesis of local spatial clustering (4, 7) and reinforcing the importance of considering geographic proximity and neighborhood effects in surveillance and control planning.

Additional variables, such as the sanitary and geographical origin of introduced animals (1, 6), the presence of grazing areas (2, 5), and the coexistence with small ruminants (2, 8), also played a relevant role, although with species-specific patterns. In particular, the buffalo cohort revealed an apparently counterintuitive result, whereby movements from non-officially free zones were associated with a lower infection risk. This outcome, possibly influenced by differences in biosecurity standards, testing intensity, or regulatory constraints in non-free zones, warrants further investigation through targeted epidemiological and molecular studies.

Overall, the study offers a comprehensive and quantitatively robust picture of the determinants influencing bTB occurrence in the regional livestock sector. The integration of epidemiological, spatial, and management data proved essential for identifying critical control points and understanding the complex transmission dynamics between farms. These insights provide valuable guidance for policymakers and veterinary authorities in refining surveillance systems, optimizing risk-based testing strategies, and promoting biosecurity awareness among farmers.

In conclusion, a coordinated, multidisciplinary approach that combines epidemiological monitoring, territorial management, and farm-level biosecurity remains crucial for effectively containing the spread of bovine tuberculosis. Strengthening these measures will not only support the long-term success of eradication programs in Campania but also contribute to improving animal health, productivity, and public health protection at the broader national level.

## Data Availability

The original contributions presented in the study are included in the article/supplementary material, further inquiries can be directed to the corresponding author.
